# Vaccination timeliness and associated factors among children aged 12–23 months in Debre Libanos district of North Shewa Zone, Oromia Regional State, Ethiopia

**DOI:** 10.3389/fped.2022.867846

**Published:** 2022-07-27

**Authors:** Hiwot Dejene, Derara Girma, Leta Adugna Geleta, Elsabeth Legesse

**Affiliations:** Department of Public Health, Salale University, Fiche, Ethiopia

**Keywords:** vaccination timeliness, Debre Libanos, North Shewa Zone, Ethiopia, children, 12-23 months

## Abstract

**Background:**

Globally, vaccination is one of the most cost-effective interventions in promoting child survival, preventing 2–3 million child deaths annually from vaccine-preventable diseases (VPDs). In Ethiopia, timely vaccination is stated as key to the prevention of unnecessary childhood mortality from measles, pneumonia, diarrheal diseases, and other VPDs. However, Ethiopia ranked fifth among the ten countries with the most unprotected children. Furthermore, previous vaccine timeliness studies produced widely disparate results. As a result, it was suggested that more research be conducted to investigate the potential factors behind the high proportion of untimely vaccination. Therefore, this study was intended to explore the association between different factors and the proportion of vaccination timeliness administered under the Expanded Program on Immunization in Debre Libanos district, Ethiopia.

**Methods:**

A community-based cross-sectional study design was employed from 1 May to 30 May 2021 among children aged 12 to 23 months with their mother/caregiver, who had started vaccination and had vaccination cards in the Debre Libanos. Simple random sampling techniques and pretested semi-structured questionnaires were used for data collection. At last, a multivariable logistic regression was used to identify factors associated with the vaccination timeliness.

**Result:**

In this study, 413 children aged 12 to 23 months were interviewed with their mother/caregiver. Overall, 33.7% [95% CI (29.1–38.3)] of children received their vaccines timely. Having a female child [AOR: 2.9, 95% CI: 1.58–5.35], mother/caregiver attending primary [AOR: 6.33, 95% CI: 2.66–15.06] and secondary/above education [AOR: 5.61, 95% CI: 2.41–13.04], sufficient vaccination knowledge [AOR: 3.46, 95% CI: 1.87–6.38], mother/caregiver with least hesitant [AOR: 3.35, 95% CI: 1.51–7.41] and middle hesitant [AOR: 1.89, 95% CI: 1.05–3.58], utilization of ANC [AOR: 2.89, 95% CI: 1.32–6.33], and giving birth at health facility [AOR: 4.32, 95% CI: 1.95–9.59] were the factors independently associated with vaccination timeliness.

**Conclusion:**

In comparison to Ethiopia’s existing vaccination coverage, the proportion of children immunized at the recommended time interval is low in the study district. Policymakers should prioritize vaccine timeliness and integrate it into childhood vaccination strategies.

## Introduction

The WHO recommends vaccines administration within a specific time frame and schedule during the first year of life (1). The administration of vaccines at the earliest acceptable age and recommended time intervals between vaccine doses are known as vaccination timeliness. Vaccinating children at an appropriate time interval is an important mechanism to develop protective antibodies to protect against diseases adequately. Immunization coverage will only translate to disease protection if an effective vaccine is administered at the appropriate times (2).

Increased adherence to vaccine timeliness protects children before exposure and reduces morbidity by increasing community immunity and limiting the spread of infectious disease, especially during disease outbreaks (3, 4). Consequently, timely vaccination is an important complementary measure to standard metrics of vaccine coverage and provides the indicators in the context of both disease control and population immunity (5). It is critical, particularly for illnesses for which most mortality occurs in the first six months of life, for example, pertussis and Haemophilus influenzae type B (Hib). Furthermore, timely vaccination promotes maximum herd immunity and protects children who are too young to be fully immunized (6).

In contrast to the aforementioned, early vaccination can fail to generate a protective antibody against the diseases (7). Because vaccinations given too soon or without a sufficient time interval between doses may not be completely protective (lead to a false sense of protection) (8). As well, delays in vaccinations also increase the risk of infection with life-threatening VPDs at the individual level (9). These will be resulted in decreasing the intervention success and reducing herd immunity at the community level (10), in completing full vaccination series (11), and increasing the risk to the resurgence of infectious diseases that are under control (12).

Globally, vaccination is one of the most cost-effective interventions in promoting child survival, preventing 2–3 million child deaths annually from vaccine-preventable diseases (VPDs) (13). In 2019, a child died every 20 s from an illness that vaccination may have averted (14). Despite the high-global vaccination coverage of 85% in 2017, some children, especially in the developing countries, face delays in obtaining routine vaccines (15). Regardless of the significance of vaccination timeliness as a public health goal for detecting adherence to vaccination schedules, this information is frequently insufficient because coverage is the most commonly used indicator (8).

Even though vaccine timeliness is an indicator of the immunization program’s quality, it has been a relatively neglected aspect of program performance (16). In line with this, globally, in low- and middle-income countries (LMICs), there is a weak supply chain management, poor access to health services, and poor service provider performance contribute to the suboptimal timeliness of vaccine schedule (17). In Sub-Saharan African (SSA) countries also, the need for country-specific further studies to clarify patterns of bottlenecks in schedule completion on the dose-specific delays (18).

In the previous studies so far, factors such as home delivery (19), low-education attainment and below four antenatal care visits (20), unplanned pregnancy and child male sex (21), highest mothers/caregivers age (22), vaccine hesitancy (5), being a multiparous mother (23), and rural children and poorest quintile (17) were independently associated with the vaccine timeliness. However, these factors are different depending on the study context (24).

In Ethiopia, vaccinating children at an appropriate time interval is the key strategy in preventing unnecessary childhood mortality from measles, pneumonia, diarrheal diseases, and other VPDs (25). However, according to the 2019 WHO/UNICEF report, Ethiopia ranked fifth among the ten countries with the most unprotected children vaccination (26).

Although it is critical for Ethiopia’s public health goal, there are few studies available to generate evidence about the untimely vaccination among children. Hence, those studies have reported a low rate of child vaccine timeliness such as 55.9% in Addis Ababa (2015) (5) and 78.1% in pastoralist areas with the CORE Group Polio Project (CGPP) intervention woredas (2015) (22). However, the studies were limited to a single residential area (5) and to pastoralist intervention woredas (22) which lacks the generalizability of evidence in a country. The results obtained were vastly discrepant from those investigations. Furthermore, the need for additional studies to identify and investigate the potential explanatory variables behind the high numbers of the untimely proportion of vaccinated in the previous studies (5, 22).

Overall, the timeliness of childhood vaccination has received close consideration in the United States and Europe (27), but in-depth investigations in low-income countries have been limited, particularly in Ethiopia. Therefore, as there had been no previous research in the Debre Libanos district of the North Shewa Zone of Central Ethiopia, the purpose of this study was to investigate the timeliness of childhood vaccination and its associated factors.

## Materials and methods

### Study design and setting

A community-based cross-sectional study was carried out in the Debre Libanos district, Central Ethiopia from May to June 2021. Debre Libanos district is located at a distance of 81.9 km to the northwest direction from the capital city of Ethiopia, Addis Ababa. The district has two urban and ten rural kebeles. The 2021 estimated number of populations in the district is 66,079. Of which 3,767 were children aged 12–23 months. In the district, there are two health centers and ten health posts that provide primary healthcare services to the community, including vaccination for children.

### Participants

All the children aged 12 to 23 months with their mother/caregiver who had started vaccination and had vaccination cards in the Debre Libanos district were the source population. Those children who had a vaccination card but no registration date of vaccination or date of birth on the card were excluded from the study.

### Sample size determination and sampling techniques

The sample size was calculated by using Epi Info STAT CALC version 7.2 with the assumptions of 95 % confidence level (CL), 0.05 margin error (d), 55.9% prevalence (P) of timely vaccinated (5), and 10% non-response rate. The final sample size was 417. Then, the sample size was allocated proportionally to the size of each kebele. At last, simple random sampling using a computer-generated random number method was used to select the study participants (i.e., mother/caregiver with their child). Then, the determined sample in each kebele was achieved through exit interviews of the mother/caregiver.

### Data collection procedures

The data were collected by using a semi-structured questionnaires. The tool was developed after reviewing different literature (1, 5, 21, 28) to estimate the magnitude of the timeliness of the vaccination among children. Moreover, the questionnaires included the sociodemographic and socioeconomic status of the respondents, knowledge and vaccination hesitancy-related questions, obstetric characteristics of the mothers, and access and health service-related factors. Data were collected using a face-to-face interview with trained 22 health professionals and supervised by 3 public health professionals. Data collectors were assigned for data collection in each kebele and supervisors have been regulated and managed the data collection process. In addition to face-to-face interviews, a chart review was done to know the timelines of vaccination.

### Measurements

**Timeliness of vaccination:** A child is considered to be timely vaccinated if the child received BCG within the first 4 weeks, OP1, Penta 1, PCV1, and Rota 1 from 6 weeks to 10 weeks, OPV 2, Penta 2, PCV 2, and Rota 2 from 14 weeks to 18 weeks, measles vaccination first dose from 9 to 10 months and for the second dose from 15 to 18 months (1, 28, 29). On the contrary, the child was considered as early vaccinated when the child received at least one dose of the vaccine below the minimum recommended age for each antigen and considered as delayed vaccination when the child received at least one dose of vaccine above the maximum recommended age (Table 1).

**TABLE 1:** National and WHO recommended vaccination timelines.

Vaccine	WHO recommendation		Operational definition	
	Minimum age	Minimum interval	Delayed	Early
BCG and OPV 0	At birth	4 weeks	>4 weeks	_
DTP-HepB1-Hib1, OPV1, PCV1, Rota1	6 weeks	4 weeks	>10 weeks	<42 days
DTP-HepB2-Hib2, OPV2, PCV2, Rota2	10 weeks	4 weeks	>14 weeks	<70 days
DTP-HepB3-Hib3, OPV3, PCV3, IPV	14 weeks	4 weeks	>18 weeks	<98 days
Measles first dose	9 months	4 weeks	>10 month	<270 days
Measles second dose	15-18 months			

**Knowledge about vaccination:** To measure knowledge on vaccination; ten knowledge questions will be used to construct a composite score. The first four questions have multiple responses and add each response from no answer to answering all the options. The rest of the six questions are based on Yes and No by giving 1 to Yes and 0 to No and selecting only one option. Based on the summation score, a score above 50% was considered as having good knowledge about childhood vaccination (5, 21).

**Vaccination hesitancy:** It was measured by the vaccination hesitancy assessing tool using ten Likert-scaled question items. Each item of the question has 5-point ranging from 1 (very unsatisfied) to 5 (very satisfied). A total score was calculated for each domain and transferred into a ‘per cent score’ by dividing the score by the possible maximum score and multiplying by 100. Based on the distribution of these sum scores, participants were categorized into three of vaccine hesitancy, dividing the sum scores evenly into the bottom third, the middle third, and the top third of hesitancy scores among mother/caregivers (30).

**Wealth index:** It was measured by a simplified and updated Ethiopian wealth index equity tool. In total, 15 questions about household assets are included in the tool. As a result, the household’s wealth index was divided into five quintiles (quintiles 1–5) and analyzed using principal component analysis. The poorest (40%) were in the first and second quintiles, the middle (20%) were in the third quintile, and the richest (40%) were in the fourth and fifth quintiles (31).

### Operational definitions and definition of terms

**Vaccination timely:** was measured if a child was vaccinated within one month after the minimum age to administer the dose as recommended by WHO (1, 28, 29) (Table 1).

**Vaccination untimely:** was measured if a child was vaccinated earlier and/or delayed than the recommended age (1, 29) (Table 1).

**Delayed vaccination:** was measured as not having received the recommended vaccine doses within one month beyond the minimum age (1, 29) (Table 1).

**Early vaccination:** doses given before the minimum age (1, 29) (Table 1).

### Data quality control

The questionnaires were translated into the local language (Afan-Oromo) and then back-translated it into English to ensure consistency. The data collectors received two days of training on the study’s objective, data gathering methods, and ethical considerations. Supervisors were also trained on how to monitor the data collection techniques. In the Girar Jarso district (adjacent to the study district), a pretest was conducted on 10% of the sample size to ensure that the questions were clear and consistent before data collection. For the actual data collection, a reliability test was performed and Cronbach’s alpha of >0.7 was used. During the data collection, supervisors verified each completed questionnaire for completeness, clarity, and consistency at the data collection location to take remedial steps.

### Data processing and analysis

Epi Data Manager version 4.41 was used to enter data, which was then exported to STATA-16 for analysis. Data were explored to assess for the completeness and descriptive statistics were employed to describe the data based on their nature. Bivariable binary logistic regression analysis was fitted on each independent variable against an outcome variable to select candidate variables at a *p*-value of ≤0.25. Then, they entered into multivariable analysis to identify factors associated with the outcome variable and to control for confounders. Model fitness was checked by the Hosmer and Lemeshow goodness-of-fit (χ^2^ = 5.466, *p*-value = 0.707). Variance inflation factor (VIF) was used to check for multicollinearity and there was no multicollinearity detected. In the multivariable binary logistic regression, a *p*-value of <0.05 with the respective adjusted odds ratio (AOR) and 95% CI was used to declare significantly associated variables.

### Ethical consideration

Ethical clearance was obtained from the Ethical Review Committee of Salale University and was given to the North Shewa zone Health Bureau. And, in turn, the permission letter was obtained from the North Shewa zone Health Bureau and the Debre Libanos Health office. The permission letter was given to Kebeles. Informed written consent was obtained from each study participant before the interview. The confidentiality was ensured.

## Results

### Socio-demographic characteristics of the respondents and the child

This study included 413 children aged 12 to 23 months who were indexed by their mother/caregiver. Approximately, 98.7% of the participants were mothers, with the remaining 1.7% caregivers. The mothers/caregivers’ mean (SD) age was 29.5 (5.96) years. Half of the respondents belonged to the age group (24–33). The majority of respondents (69.7%) were rural residents. About two-thirds (67.1%) were married, and more than a quarter (26.9%) lack formal education. Regarding the children’s characteristics, the mean (SD) age of the children in months was 16.1 (3.1). Approximately 40.9% of the children were males, and one-third were born in the spring and winter seasons (Table 2).

**TABLE 2:** Sociodemographic characteristics of the respondents and the child for vaccination timeliness and associated factors among children aged 12–23 months in Debre Libanos district North Shewa Zone, Oromia Regional State, Ethiopia 2021.

Variables	Categories	Frequency	Percentage
Sex of the child	Male	169	40.9
	Female	244	59.1
Residence	Urban	125	30.3
	Rural	288	69.7
Mother/caregiver age	15-24	48	11.6
	25-35	220	53.3
	> 35	145	35.1
Marital status of the mother/caregiver	Married	277	67.1
	Divorced	87	21.1
	Single	49	11.9
Educational status of mother/caregiver	No formal education	111	26.9
	Primary education (1-8)	150	36.3
	Secondary and higher education	152	36.8
Educational status of the father	No formal education	38	13.7
	Primary education (1-8)	120	43.3
	Secondary and higher education	119	43.0
Occupational status of mother/caregiver	Housewife	134	32.4
	Farmer	145	36.1
	Employed {government/non-government}	114	26.7
	Merchant	20	4.8
Occupational status of the father	Farmer	119	43
	Employed {government/non-government}	81	19.6
	Merchant	77	18.6
Birth season of the child	Summer	68	16.5
	Autumn	104	25.2
	Spring	120	29.1
	Winter	121	29.3
Birth order of the child	1	122	29.5
	2-4	228	55.2
	≥ 5	63	15.3
Household wealth index	Lowest wealth index	130	31.5
	Middle wealth index	125	30.3
	Highest wealth index	158	38.2

### Obstetric-related factors

The majority of respondents, 334 (80.9%), had at least one pregnancy or more. The average (SD) number of pregnancies per woman was 3 (1.0). More than three-quarters of the previous pregnancy status was planned, and the majority of respondents had ANC visits for the previous child pregnancy. More than two-thirds of the participants (78.9%) had their current child at the health facility, and the majority (76.5%) used the postnatal care service (Table 3).

**TABLE 3:** Obstetric related factors for vaccination timeliness and associated factors among children aged 12–23 months in Debre Libanos district North Shewa Zone, Oromia Regional State, Ethiopia 2021.

Variables	Categories	Number	Percentage %
Number of pregnancies	Primigravida	54	13.1
	Multigravida	334	80.9
	Grand multigravida	25	6.0
Number of alive children	1 child	127	30.8
	2-4 children	214	51.8
	> = 5 children	72	17.4
Last pregnancy status	Planned	330	79.9
	Unplanned	83	20.1
ANC visit	Yes	331	80.1
	No	82	19.9
Number of ANC visit	1	129	38.9
	2	144	43.5
	≥ 3	58	17.6
TT dose received during the pregnancy	No	72	17.4
	One	194	47.0
	Two or more	147	35.6
PNC service utilization	Yes	326	78.9
	No	87	21.1
Place of delivery	At health facility	316	76.5
	Home	97	23.5

### Mother-related factors

**FIGURE 1:**
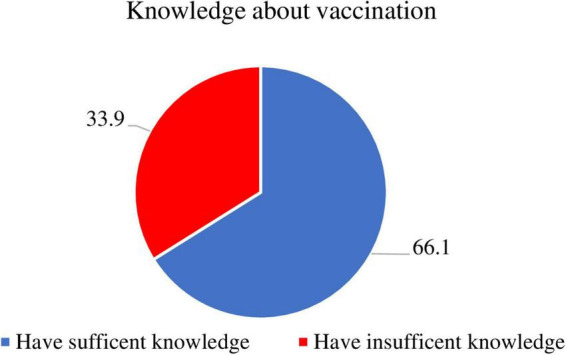
Knowledge about vaccination among mothers/caregivers.

About two-thirds of the participants (66.1%) had sufficient knowledge about vaccination. Figure 1: knowledge about vaccination among mothers/caregivers.

**FIGURE 2:**
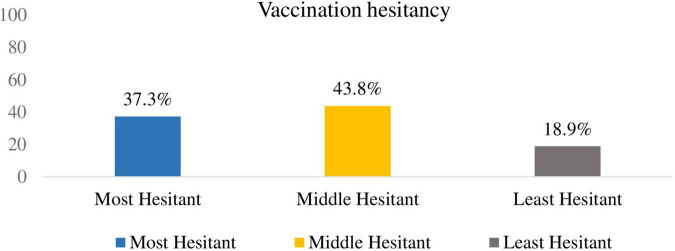
Vaccination hesitancy among mothers/caregivers.

About half (43.8%) of the respondents were middle hesitant about vaccination followed by the most hesitant, which accounts for (37.3%) (Figure 2: vaccination hesitancy among mothers/caregivers).

### Access-related factors

About 59.3% of the respondents took less than 30 min to go to the vaccination site and around 40.9% of the respondents did not use transportation to get to the vaccination site. Nearly, two-thirds of the participants get information about vaccination from health extension workers (Table 4).

**TABLE 4:** Access-related factors for vaccination timeliness and associated factors among children aged 12–23 months in Debre Libanos district North Shewa Zone, Oromia Regional State, Ethiopia 2021.

Variables	Categories	Number	Percentage %
Time taken to vaccination site	< 30 min	245	59.3
	≥ 30 min	168	40.7
Mode of transportation	On foot	169	40.9
	By vehicle	32	7.7
	By bus	31	7.5
	By cart/ animal	181	43.8
Source of information	Mobile	67	16.2
	Television	35	8.5
	Radio	43	10.4
	Health extension worker	268	64.9
Place of vaccination received	Health center	214	51.8
	Health post	199	48.2

### Vaccination timeliness

**FIGURE 3:**
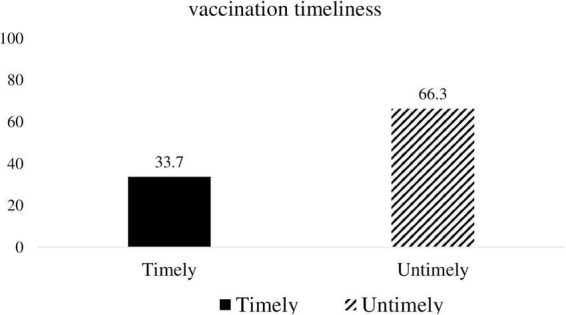
Vaccination timeliness among children aged 12-23 months.

Overall, 33.7% [95% CI: 29.1–38.3] of the children received their vaccinations at the recommended time interval. Of the total 66.3% of children who did not receive vaccinations at the recommended interval, 25.5% [95% CI: 20.9–30.1] and 74.5% [95% CI: 69.9–79.1] received their vaccinations earlier and later than the recommended time interval, respectively (Figure 3: vaccination timeliness among children aged 12–23 months).

### Vaccination timeliness for specific vaccines

**FIGURE 4:**
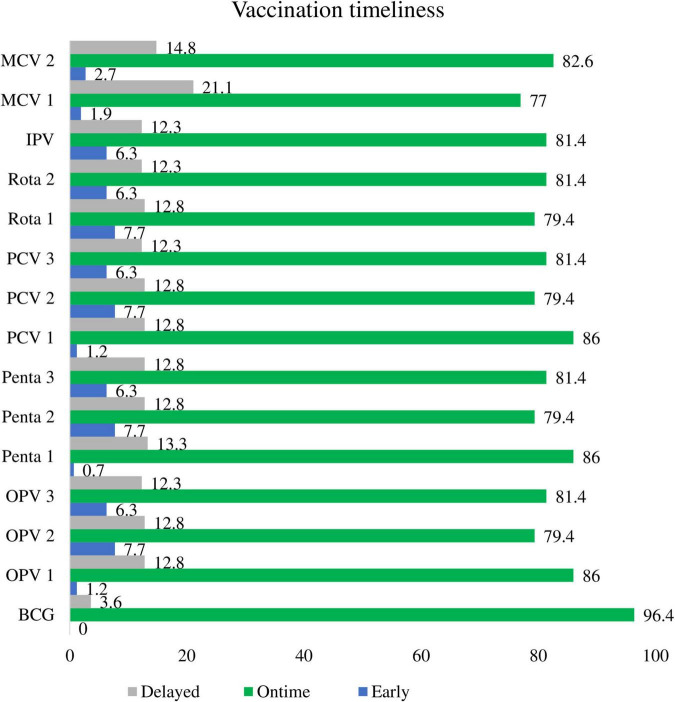
Vaccination timeliness for specific antigen among children aged 12-23 months.

About 96.4, 86.0, 81.4, and 77.0% of children received BCG, Penta1, Penta3, and measles vaccines at the recommended time interval, respectively. Moreover, 0.7, 6.3, and 1.9% of children received Penta1, Penta3, and measles vaccines earlier than the acceptable time interval, respectively. On the contrary, 3.6, 13.3, 12.3, and 21.1% of children took BCG, Penta1, Penta3, and measles vaccines later than the acceptable time interval, correspondingly (Figure 4: vaccination timeliness for specific antigen among children aged 12–23 months).

### Perceived reasons for not vaccinating children timely

**FIGURE 5:**
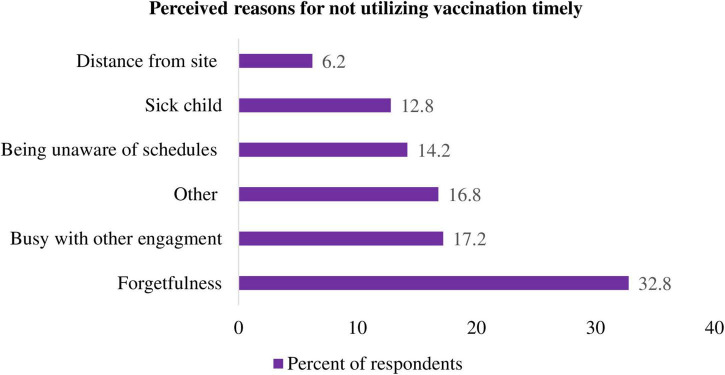
Perceived reasons for not vaccinating children timely.

The reasons given by mothers/caregivers for not attending vaccination schedules timely were 32.8% forgetfulness, 17.2% being busy with other commitments, and the rest being unaware of the schedule, being distant from the site, and the child being sick at the time of the vaccine schedule (Figure 5: perceived reasons for not vaccinating children timely).

### Factors associated with vaccination timeliness

In the bivariable logistic regression analysis: a place of residence, sex of the child, educational status of the mother/caregiver, marital status of the mother/caregiver, birth order, knowledge about vaccination, vaccination hesitancy, number of alive children, last pregnancy status, utilization of ANC, utilization of TT dose, place of delivery, and utilization of PNC were candidates for multivariable logistic analysis.

In multivariable logistic regression analysis, variables such as sex of the child, educational status of the mother/caregiver, knowledge about vaccination, vaccination hesitancy, utilization of ANC, and place of delivery were independently associated with vaccination timeliness.

Accordingly, having a female child was found two times [AOR: 2.9, 95% CI: 1.58–5.35] more likely to get vaccination on time than having a male child. Mother/caregiver who attended primary and secondary and above education was six [AOR: 6.33, 95% CI: 2.66–15.06] and five times [AOR: 5.61, 95% CI: 2.41–13.04] more likely to vaccinate their children timely compared with the mother/caregiver with no formal education, respectively.

A mother/caregiver having sufficient knowledge about vaccination was found to be three times more likely to vaccinate their children timely than a mother/caregiver having insufficient knowledge. [AOR: 3.46, 95% CI: 1.87–6.38] and also a mother/caregiver with the least hesitant and middle hesitant three [AOR: 3.35, 95% CI: 1.51–7.41] and two [AOR: 1.89, 95% CI: 1.05–3.58] times more likely to vaccinate their child timely than the most hesitant, respectively.

The utilization of ANC was another factor that affected the timeliness of vaccination. A mother/ caregiver who utilized ANC was three times more likely to vaccinate their child within the recommended time interval than her counterparts. [AOR: 2.89, 95% CI: 1.32–6.33]. Similarly, place of delivery was an independent factor associated with the timeliness of vaccination. A mother who delivered child in a health facility increases the rate of vaccinating her child within the recommended time interval by four times than a mother who delivered the child at home [AOR: 4.32, 95% CI: 1.95–9.59] (Table 5).

**TABLE 5:** Factors associated with vaccination timeliness among children aged 12–23 months in Debre Libanos district, North Shewa Zone, Oromia Region State, Ethiopia 2021.

Variable	Categories	Vaccination timeliness		COR (95% CI)	AOR (95% CI)
		Timely n (%)	Untimely n (%)		
Residence	Urban	54 (43.2)	71 (56.8)	Ref	Ref
	Rural	85 (29.5)	203 (70.5)	1.82 [1.17-2.81]	1.34 [0.70-2.58]
Sex of the child	Male	29 (17.2)	140 (82.8)	Ref	Ref
	Female	110 (45.1)	134 (54.9)	3.96 [2.47-6.35]	2.91 [1.58-5.35] **
Educational status of the mother/caregiver	No formal education	13 (11.7)	98 (88.3)	Ref	Ref
	Primary	65 (43.3)	85 (56.7)	5.76 [2.97-11.18]	6.33 [2.66-15.06] *
	Secondary and above	61 (40.1)	91 (59.9)	5.05 [2.60-9.81]	5.61 [2.41-13.04] *
Marital status of the mother/caregiver	Married	120 (43.3)	157 (56.7)	3.39 [1.58-7.27]	2.73 [0.99-7.49]
	Divorced	10 (11.5)	77 (88.5)	0.57 [0.22-1.54]	0.36 [0.59-1.38]
	Single	9 (18.4)	40 (81.6)	Ref	Ref
Birth order	1	60 (47.2)	67 (52.8)	3.07 [1.45-6.46]	0.48 [0.10-2.27]
	2-4	57 (26.6)	157 (73.4)	2.55 [1.26-5.17]	2.81 [0.99-17.83]
	≥ 5	22(30.6)	50 (69.4)	Ref	Ref
Knowledge about vaccination	Sufficient knowledge	109 (39.9)	164 (60.1)	2.44 [1.52-3.90]	3.46 [1.87-6.38] **
	Insufficient knowledge	30 (21.4)	110 (78.6)	Ref	Ref
Vaccination hesitancy	Most hesitant	32 (20.8)	122 (79.2)	Ref	Ref
	Middle hesitant	70 (38.7)	111 (61.3)	2.40 [1.47-3.92]	1.89 [1.05-3.58] *
	Least hesitant	37 (47.4)	41 (52.6)	3.44 [1.91-6.21]	3.35 [1.51-7.41] **
Number of alive children	1 child	60 (47.2)	67 (52.8)	2.04 [1.11-3.74]	3.47 [0.26-9.51]
	2-4 children	57 (26.6)	157 (73.4)	0.83 [0.46-1.48]	0.31 [0.13-0.73]
	> = 5 children	22(30.6)	50 (69.4)	Ref	Ref
Last pregnancy status	Planned	128 (38.8)	202 (61.2)	4.15 [2.12-8.12]	3.71 [0.63-5.42]
	Unplanned	11 (13.3)	72 (86.7)	Ref	Ref
Utilization of ANC	Yes	123 (37.2)	208 (62.8)	2.44 [1.35-4.40]	2.89 [1.32-6.33] **
	No	16 (19.5)	66 (80.5)	Ref	Ref
Utilization of TT dose	No	20 (27.8)	52 (72.2)	Ref	Ref
	1 dose only	60 (30.9)	134 (69.1)	1.16 [0.64-2.12]	1.22 [0.54-2.78]
	2 and more dose	59 (40.1)	88 (59.9)	1.74 [0.95-3.22]	1.73 [0.74-4.05]
Place of delivery	Home	22 (22.7)	75 (77.3)	Ref	Ref
	At health facility	117 (37.0)	199 (63.0)	2.00 [1.18-3.39]	4.32 [1.95-9.59] *
Utilization of PNC	Yes	123 (37.7)	203 (62.3)	2.69 [1.49-4.84]	2.23 [0.76-6.51]
	No	16 (18.4)	71 (81.6)	Ref	Ref

*Statistically significant at P-value of ≤0.05, **statistically significant at P-value of <0.001.

## Discussion

This study measures the magnitude and associated factors of vaccination timeliness among children aged 12–23 months. Accordingly, 33.7% of the children received their recommended vaccination timely. The study from Gondar city, north-west Ethiopia reported a consistent finding of 31.9% (32). A similar population age group, sampling technique, and use of an outcome ascertainment tool may result in consistent findings. However, this finding is higher than 6.2% reported in Menz Lalo district of Northeast Ethiopia (21) and 23.9% in Toke Kutaye district, central Ethiopia (28). This disparity could be attributed to variations in study approach, location, healthcare access, and study period.

In contrast, this finding is below the Ethiopian DHS 2019 report of 40% (33). The difference could be attributed to sample size, sampling methods, and geographical area coverage. Moreover, this study is lower than a study from Addis Ababa, Ethiopia that showed vaccination timeliness of 55.9% (5). This could be because the current study was conducted among children living in rural areas and is a community-based study, as opposed to the Addis Ababa study.

Having a female child increased the likelihood of receiving vaccinations timely. Comparable finding was reported from Senegal (34). This could be encouraged in the rural community to maintain equality and a positive attitude toward avoiding child sex preferences. Because, in Ethiopia, there is a sex preference for male child in terms of timely vaccination (21).

A mother/caregiver who attends formal education is more likely to vaccinate her child at the recommended time interval. This finding is comparable with the studies done in Ethiopia (32), India (16), and Iran (35) indicates that mother/caregiver’s attending formal education reduce the risk of untimely child vaccinating. This is because a higher education level can facilitate the mother’s/caregiver’s communication with health workers, influencing their awareness of seeking and utilizing public health services such as child vaccination (32).

Moreover, having sufficient knowledge about vaccination increases the odds of vaccinating the child at the recommended schedule. Similarly, studies done in the northeast Ethiopia (21) and central Ethiopia (28)showed that insufficient knowledge about vaccination increased the delay in vaccinating at the recommended interval. The possible explanation is that knowledge lessens the likelihood of having negative feelings about childhood vaccination, which increases practice and timeliness. Also, knowing the vaccination schedule, VPDs, and reasons for vaccination will increase the likelihood of vaccinating children on time (28). As well, vaccine hesitancy significantly increased the odds of untimely vaccination. This figure is supported by a study done in Addis Ababa (5). This could be because if the mother’s/caregiver’s were more hesitant about the vaccine, it would increase the delay in vaccine acceptance/refusal.

In the current study, the use of ANC was realized to be another factor that increases vaccination timeliness. A study conducted in northeast (32) and northwest (32) Ethiopia found that ANC utilization have decreased vaccination delays. In this study, giving birth at a health facility was also significantly associated with vaccine timelines. This finding is supported by studies conducted in Ethiopia by analyzing EDHS data (36) and Kutaye district (28), which revealed that if the mother delivers the child at a health facility, it increases the timely initiation of vaccination at the recommended interval. This is because mothers who delivers the child in a health facility had a greater opportunity of being advised about the benefits of EPI services and getting health education (28).

### Limitation of the study

The cross-sectional nature of the study design does not allow causality ascertainment. The study participants were selected based on the presence of immunization cards, which might lead to selection bias because infants whose parents did not keep their immunization cards were excluded from the study.

## Conclusion

The proportion of children vaccinated at the recommended time interval is low in the study area as compared to the current performance of the vaccination coverage in Ethiopia. Not all children aged 12–23 months in the study area were vaccinated with their recommended vaccine at the schedule. The factors that increase the likelihood of timely vaccination of children were mother/caregiver attended primary and above education level, having female sex child, sufficient knowledge about vaccination, middle and less hesitant for a vaccine, utilization of ANC, and giving birth at the health facility. Therefore, in order to adhere to the recommended schedule, mothers/caregivers should receive prompt attention on the identified factors through a plausible program. Furthermore, to improve children’s immunological wellbeing, policymakers should emphasize and incorporate vaccine timeliness monitoring indicators into childhood vaccination strategies.

## Data availability statement

The raw data supporting the conclusions of this article will be made available by the authors, without undue reservation.

## Ethics statement

The studies involving human participants were reviewed and approved by Salale University Ethics Committee. The patients/participants provided their written informed consent to participate in this study.

## Author contributions

HD made substantial contributions to conception and design, acquisition of data, and analysis and interpretation of data. DG, LG, and EL were involved in the analysis, interpretation of data, and drafting the manuscript or revising it critically for important intellectual content. All authors read and approved the final manuscript.

## Conflict of interest

The authors declare that the research was conducted in the absence of any commercial or financial relationships that could be construed as a potential conflict of interest.

## Publisher’s note

All claims expressed in this article are solely those of the authors and do not necessarily represent those of their affiliated organizations, or those of the publisher, the editors and the reviewers. Any product that may be evaluated in this article, or claim that may be made by its manufacturer, is not guaranteed or endorsed by the publisher.
